# An sEMG-Based Human-Exoskeleton Interface Fusing Convolutional Neural Networks With Hand-Crafted Features

**DOI:** 10.3389/fnbot.2022.938345

**Published:** 2022-07-01

**Authors:** Xiao Yang, Zhe Fu, Bing Li, Jun Liu

**Affiliations:** ^1^Graduate School of Tianjin Medical University, Tianjin, China; ^2^Joint Department, Tianjin Hospital, Tianjin Medical University, Tianjin, China

**Keywords:** human-robot interfaces, surface electromyography, lower limb movement prediction, feature fusion, hemiplegia rehabilitation training

## Abstract

In recent years, the human-robot interfaces (HRIs) based on surface electromyography (sEMG) have been widely used in lower-limb exoskeleton robots for movement prediction during rehabilitation training for patients with hemiplegia. However, accurate and efficient lower-limb movement prediction for patients with hemiplegia remains a challenge due to complex movement information and individual differences. Traditional movement prediction methods usually use hand-crafted features, which are computationally cheap but can only extract some shallow heuristic information. Deep learning-based methods have a stronger feature expression ability, but it is easy to fall into the dilemma of local features, resulting in poor generalization performance of the method. In this article, a human-exoskeleton interface fusing convolutional neural networks with hand-crafted features is proposed. On the basis of our previous study, a lower-limb movement prediction framework (HCSNet) in patients with hemiplegia is constructed by fusing time and frequency domain hand-crafted features and channel synergy learning-based features. An sEMG data acquisition experiment is designed to compare and analyze the effectiveness of HCSNet. Experimental results show that the method can achieve 95.93 and 90.37% prediction accuracy in both within-subject and cross-subject cases, respectively. Compared with related lower-limb movement prediction methods, the proposed method has better prediction performance.

## 1. Introduction

With the development of artificial intelligence and robotics, lower-limb exoskeleton robots have become a hot spot in the field of medical rehabilitation. It has played a significant role in the rehabilitation training of patients with hemiplegia (Zhuang et al., 2020; Calafiore et al., 2021). Through rehabilitation training with exoskeleton robots rather than traditional therapists, positive outcomes have been reported (Krebs et al., 1998; Grimaldi and Manto, 2013; Jarrassé et al., 2014). The lower limb exoskeleton robot realizes the perception and prediction of the lower limb movements of patients with hemiplegia through the HRI. It then drives the motor to assist the patient in completing the movement and achieve the effect of rehabilitation training (Huang et al., 2015; Calafiore et al., 2021).

Traditional HRI mainly uses physical sensors, such as crutches and force sensors, to directly predict the lower limb movements of patients with hemiplegia (Yan et al., 2017; Solanki and Lahiri, 2018; Nozaki and Watanabe, 2019). This kind of HRI is simple and effective, but it is motion-lag, i.e., patients with hemiplegia must perform lower limb movements before HRI can perceive them and perform corresponding movements. In recent years, with the decoding of biological signals, HRI based on biological signals (such as electroencephalogram and electromyography) have been designed, opening up the possibility of realizing more efficient and accurate lower limb movement prediction (Suplino et al., 2019; Ortiz et al., 2020).

Human-robot interfaces based on biological signals is mainly divided into EEG-based HRI and sEMG-based HRI. Compared with EEG signals, sEMG signals have a higher signal-to-noise ratio and are less susceptible to interference from environmental factors. In addition, sEMG signals are usually generated within 30–500 ms before limb movement and are the physiological signals most relevant to limb movement (Reaz et al., 2006). Therefore, HRI based on sEMG signals is earlier and more widely applied to lower-limb exoskeletons. sEMG-based HRI consists of three main processing stages (Li et al., 2020): data collection and processing stage, where sEMG data is recorded and preprocessed; feature extraction stage, where meaningful information is extracted from the sEMG data; and classification stage, where a motion intention is interpreted from the data. Many related studies have shown that feature extraction is crucial for HRI movement prediction, and it determines the upper limit of the prediction accuracy (Phinyomark et al., 2012; Samuel et al., 2018).

Traditional sEMG-based HRI usually extracts hand-crafted features and then uses machine learning methods to build mappings of hand-crafted features and different lower limb movements (Jose et al., 2017; Motoche and Benalcázar, 2018; Narayan et al., 2018; Khiabani and Ahmadi, 2021; Zhou et al., 2021). The interface is computationally cheap and can achieve a relatively good lower-limb movements prediction performance in most cases. Since the hand-crafted features only contain some superficial heuristic information, the sEMG signals of different patients with hemiplegia during lower-limb movements vary greatly. Therefore, it is difficult and time-consuming to achieve efficient and accurate lower-limb movement prediction for different patients with hemiplegia. Deep learning has largely alleviated the need for manual feature extraction, achieving state-of-the-art performance in fields such as computer vision (Hinton et al., 2012). In fact, deep convolutional neural networks (CNNs) can automatically extract appropriate features from the data. Some researchers have designed deep learning-based HRIs, which can achieve higher lower limb movement prediction performance than hand-crafted features (Atzori et al., 2016; Hartwell et al., 2018; Duan et al., 2019; Burns et al., 2020). However, due to the large amount of data required by deep learning, when predicting small datasets (such as the lower extremity motion dataset for a single hemiplegic patient), the lower-limb movement prediction method based on deep learning often suffers from overfitting. This results in poor movement prediction performance and low generalization ability of the model.

Aiming at the above problems, this article proposes a human-exoskeleton interface for lower limb movement prediction in patients with hemiplegia. Building on our previous study (Shi et al., 2021), this interface builds a lower-limb movement prediction framework (HCSNet) which can extract the time and frequency domain hand-crafted features and channel synergy learning-based features. By introducing a channel attention mechanism to deeply fuse handcrafted features and learning-based features, efficient and accurate lower-limb movement prediction is achieved. The main contributions of this article are shown as follows:

- An sEMG-based HRI fusing convolutional neural networks with hand-crafted features is proposed for lower limb movement prediction of patients with hemiplegia. It uses the sEMG signals of the unaffected lower limbs of patients with hemiplegia to predict lower-limb movements and combines the time-frequency domain hand-crafted features and the channel synergy learning-based features extracted by MCSNet, which improves the accuracy of lower limb movement prediction.
- An sEMG data acquisition experiment is designed to verify the proposed lower-limb movement prediction framework (HCSNet). In the two cases of within-subject and cross-subject, the lower limb movement prediction performance of HCSNet is compared with four traditional machine learning-based movement prediction models and three deep learning-based movement prediction models.

## 2. Related Study

Human-robot interfaces is widely used in lower-limb exoskeleton robots for movement prediction of hemiplegia patients during rehabilitation training. It can be divided into physical signal-based HRI and sEMG-based HRI.

### 2.1. Physical Signal-Based HRI Related Study

Physical signals used for HRI generally include crutches button command, inertial measurement units (IMU) or pressure signals. Yan et al. developed a cane-type walking-aid robot and proposed a fusion prediction method based on the cane-type walking-aid robot for the coordinated movement of upper and lower limbs. The results show that the method is effective in reality (Yan et al., 2017). Nozaki et al. developed a method to estimate the stride length of patients during walking by using IMU attached to the feet and used an artificial neural network to automatically detect the movement state of a hemiplegic gait. The results proved that the artificial neural network with a feature extraction layer can effectively detect the movement state of hemiplegic subjects (Nozaki and Watanabe, 2019). Solanki et al. designed a pair of shoes containing a force sensing resistor (FSR) and a wireless data acquisition unit. Real-time FSR data was transmitted to the console for analysis through wireless mode, realizing the measurement and prediction of patient step characteristics (Solanki and Lahiri, 2018).

Physical signal-based HRI is simple and effective, but it is motion-lag. In addition, with the increase of interactive instructions, the interaction complexity of the physical signal-based HRI will greatly increase, bringing a tremendous cognitive load to patients with hemiplegia. With the continuous development of biological neural engineering and brain science, HRI represented by electromyography and other biological signals gradually appear in people's vision and are applied in lower-limb exoskeleton robots to predict patients' movements.

### 2.2. sEMG-Based HRI Related Study

As the biological signal most relevant to exercise, sEMG has been applied to HRI for a long time, and the research on sEMG-based HRI is particularly rich.

A complete sEMG-based HRI process includes data acquisition and processing, feature extraction, and feature classification. Traditional prediction methods usually use hand-crafted features and then use machine learning methods to construct mappings between features and movements. Motoche et al. proposed a classification model based on sEMG. The preprocessed signal values and the results of a set of functions were selected as the extracted features, and the features were classified by an artificial neural network (ANN). The accuracy of the classification model reached 90.7% (Motoche and Benalcázar, 2018). Jose et al. (2017) extracted the time domain sEMG features of the subjects' forearm movement and classified the features using a multi-layer perception network, with a classification accuracy of 91.6%. In the literature (Zhou et al., 2021), the machine learning method was applied to the recognition of shoulder movements, and the support vector machine (SVM) method with a sliding time window of 270ms was used, and the classification accuracy was more than 90% (Hinton et al., 2012). Narayan et al. (2018) extracted the features of the sEMG signal by first-order differential and classified the features by a medium tree classifier, which improved the classification accuracy by 6% compared with other features.

Recent research has explored the application of deep learning in HRI. Burns et al. proposes a classification method combining discrete wavelet transform and enhanced probabilistic neural network (EPNN). Compared with SVM, k-Nearest Neighbor (KNN), and probabilistic neural network, it is proved that the performance of the proposed method is better than that of machine learning algorithm alone (Burns et al., 2020). Atzori et al. (2016) applied convolutional neural network to sEMG data classification, and the proposed framework classification accuracy was higher than the average accuracy obtained by classical methods, with the highest accuracy reaching 87.8%. In the literature (Hartwell et al., 2018), a compact deep neural network architecture is used, which still achieves a classification accuracy of 84.2% even though the parameter values of other networks are several orders of magnitude less. Duan et al. (2019) applied multi-channel convolutional neural network to sEMG dataset for gesture recognition, and the recognition accuracy was 90%.

Most of the previous study has focused on utilizing sEMG signals from the bilateral limbs of hemiplegic patients. For patients with hemiplegia, the sEMG signal of the affected limb is weak, and the problems of muscle associated reactions and abnormal discharge are prone to occur (Lindsay et al., 2019). Furthermore, most of the previous studies use deep convolutional networks to extract the learning-based features from data, ignoring the heuristic information contained in hand-crafted features.

### 2.3. Application of HRI on Exoskeleton

The exoskeleton is a typical scenario of HRI applications. The application of HRI in exoskeletons can be divided into movement prediction (Kyeong et al., 2019; Read et al., 2020) and state detection (Bae et al., 2019). Movement prediction refers to predicting the limb movement of the exoskeleton wearer. Kyeong et al. (2019) designed a hybrid HRI based on sEMG and foot pressure signals to predict the wearer's gait cycle. Literature (Read et al., 2020) applied HRI based on the wearer's IMU signals and crutches pressure measurement signals to the Ekso exoskeleton. It helps the Ekso exoskeleton predict the wearer's standing and walking movements. State detection is to observe the physiological state of the exoskeleton wearer during using the exoskeleton. Bae et al. (2019) designed an HRI to help observe whether the wearer experiences spasms when using the exoskeleton.

This article is mainly to design an HRI based on sEMG and apply it to a lower-limb exoskeleton robot for movement prediction of patients with hemiplegia during rehabilitation training. It is not the same as HRI based on hand-crafted features and learning-based features. Based on our previous study (Shi et al., 2021), we construct a lower limb movement prediction framework (HCSNet) that fuses hand-crafted features and deep neural networks. The synergy features of different sEMG channels are extracted using MCSNet, which are subsequently combined with hand-crafted features such as time and frequency domains. Through the heuristic information of hand-crafted features and the abstract and personalized information of learning-based features, the accuracy of HCSNet's lower-limb movement prediction for patients with hemiplegia is improved.

## 3. Methods

This section mainly introduces the proposed lower-limb movement prediction model. Section 3.1 describes the overall architecture and methodology details of the HCSNet model. In Section 3.2, We introduce several commonly used lower-limb movement prediction models to compare and analyze the effectiveness of the proposed HCSNet.

### 3.1. Description of the HCSNet Model

#### 3.1.1. Overview of the HCSNet Model

**Figure 1:**
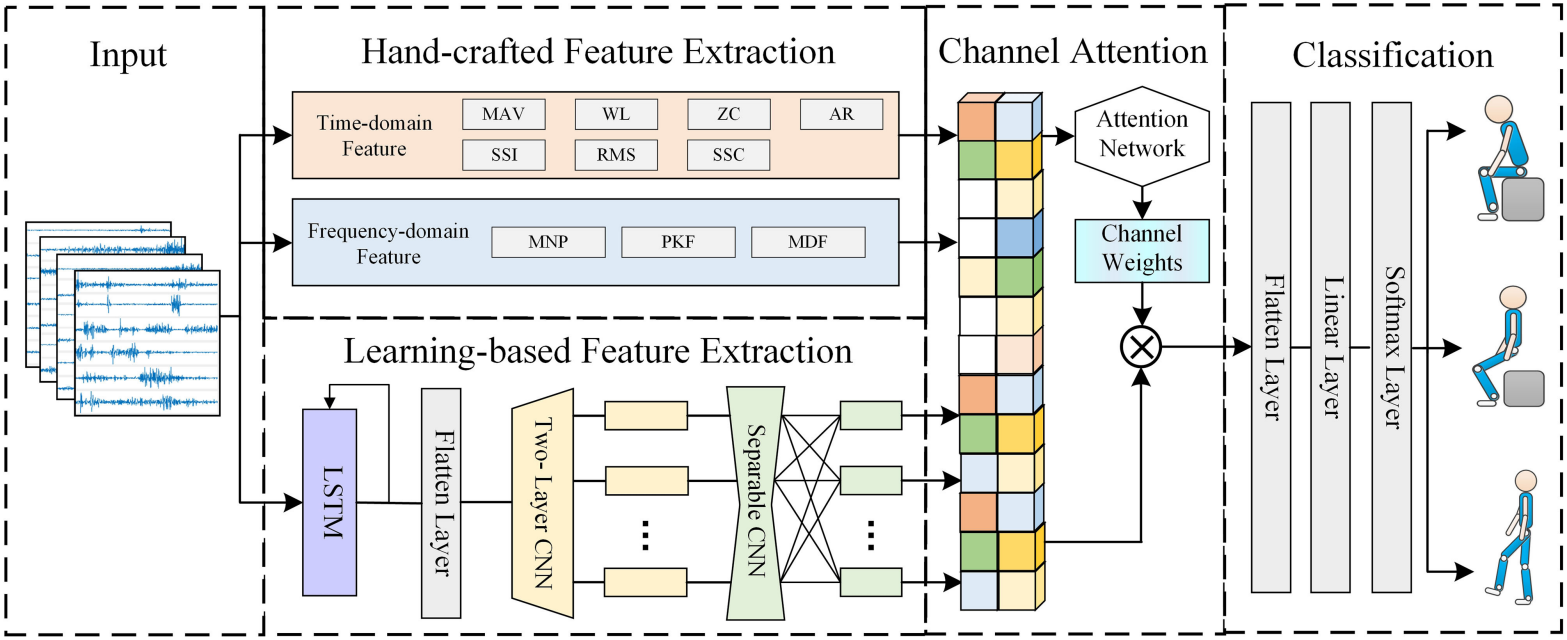
The overall architecture of the HCSNet model. HCSNet is divided into three parts. The first part is data input, and the second part is feature extraction, which uses traditional methods and our previous work MCSNet to extract hand-crafted features and learning-based features respectively, and then uses the attention mechanism to fuse the hand-crafted and learning-based features. The third part is movement prediction/classification.

Figure 1 depicts the overall framework of HCSNet. It can be clearly seen that HCSNet is divided into three parts. The first part is data input, inputting the divided lower-limb sEMG signals. The second part is feature extraction, which uses traditional methods and our previous study MCSNet (Shi et al., 2021) to extract hand-crafted features and learning-based features respectively, and then uses the attention mechanism to fuse the hand-crafted and learning-based features. The third part is movement prediction/classification, which classifies the fusion features extracted in the second part. The sEMG signal has *C* channels and *T* time sample points, which means that $X_{sEMG}\in {ℜ}^{C\times T}$.

#### 3.1.2. Hand-Crafted Feature Extraction

For the input sEMG signal *X*_*sEMG*_, we extract hand-crafted features from two dimensions, time domain and frequency domain, so as to obtain the heuristic information contained in the signal. Referring to the research conclusions of time domain and frequency domain features in the literature (Phinyomark et al., 2012), we finally selected 7 time-domain features and 3 frequency-domain features. The selected time domain features are Mean Absolute Value (MAV), Simple Square Integral (SSI), Waveform Lengths (WL), Root Mean Squared (RMS), Zero Crossings (ZC), Slope Sign Changes (SSC), and 6-order Auto-Regressive coefficient (6-AR). The selected frequency domain features are Mean Power (MNP), Peak Frequency (PKF), and Median Frequency (MDF). The extracted time-domain features and frequency-domain features are expressed as $F_{td}\in {ℜ}^{1\times 7*C}$ and $F_{fd}\in {ℜ}^{1\times 3*C}$.

#### 3.1.3. Learning-Based Feature Extraction

For learning-based features, this article adopts the previous work MCSNet to extract. MCSNet is a deep learning-based lower limb movements prediction model, which contains three modules in total. The first module is an LSTM layer, which is used to extract the temporal features of the sEMG signal channel by channel. The second module is a two-layer CNN network. It extracts the temporal-frequency features of different channels of the sEMG signal. The third module of MCSNet is a Depthwise CNN layer, which is used to combine and optimize the temporal-frequency features between different channels. Namely, it is used to extract the synergy features between the sEMG signal channels. The learning-based feature *F*_*learning*_ extracted by MCSNet can be expressed as:


(1) $$\begin{array}{r} F_{learning}=MCSNet\left(X_{sEMG}\right), \end{array}$$

#### 3.1.4. Feature Fusion Based on Channel Attention Mechanism

After acquiring hand-crafted features and learning-based features, we introduce a channel attention module for fusion between different features. The hand-crafted features contain heuristic information in the time and frequency domains of sEMG signals, and the learning-based features contain the synergy information between different sEMG feature channels. The channel attention module can learn the weights of different features to fuse different feature information, improving the model's performance for lower limb movement prediction. The operation of the channel attention module can be described as:


(2) $$\begin{array}{l} \;F_{fusion}=[F_{td},F_{fd},F_{learning}],\;\\ W_{channel}(F_{fusion})=\sigma (MLP(AvgPool(F_{fusion}))\;\\ \;+MLP(MaxPool(F_{fusion}))),\;\\ \;F=W_{channel}(F_{fusion})⊗F_{fusion}, \end{array}$$

Among them, *F*_*fusion*_ is the combined feature of hand-crafted features and learning-based features, σ is a non-linear function, *W*_*channel*_ is the weight of different feature channels, and *F* is the final fusion feature, ⊗ represents matrix multiplication.

After obtaining the fused features, we flatten the features and then feed them into a linear layer and softmax layer, and finally get the predicted lower limb movements. This series of operations can be expressed as:


(3) $$\begin{array}{r} Label_{predicted}=Softmax\left(linear\left(F\right)\right). \end{array}$$

### 3.2. Comparison With Other Movement Prediction Approaches

We compare HCSNet with four classic lower limb movement prediction methods based on hand-crafted features and machine learning and three state-of-the-art deep learning-based lower limb movement prediction methods. By analyzing different methods in both within-subject and cross-subject situations, the effectiveness of HCSNet is verified.

For traditional lower limb motion prediction methods based on hand-crafted features and machine learning, we use the same hand-crafted features as HCSNet to ensure the objectivity of the analysis results. In terms of classification algorithms, after investigating several pieces of literature, we finally selected four classification algorithms: Linear Discriminant Analysis (LDA), Radial Basis Function-Based Support Vector Machine (RBFSVM), K-Nearest Neighbors (KNN), and Random Forest (RF).

Among the deep learning algorithms, we compare HCSNet with three state-of-the-art lower limb movement prediction algorithms: MCSNet, GoogleNet, and ResNet. MCSNet is our previous study (Shi et al., 2021), which extracts synergy features between different sEMG signal channels by fusing LSTM and attention mechanisms. GoogleNet (Szegedy et al., 2015) and ResNet (Li et al., 2018) are two very popular deep learning algorithms in the field of computer vision. They can extract deeper features through some special structures. This article uses GoogleNet V2 and ResNet16.

## 4. Experiments and Results

In this part, an sEMG signal acquisition experiment is designed to verify the effectiveness of the method proposed in this article. Section 4.1 describes the sEMG acquisition experimental paradigm and data preprocessing method. Section 4.2 gives the implementation details of model training. In Section 4.3, We compare the movement prediction performance between HCSNet and other movement prediction models in the case of within-subject and cross-subject.

### 4.1. sEMG Data Acquisition Experiment

A total of 10 healthy subjects were invited to participate in the experiment. Each subject randomly completed three movements of standing, sitting, and walking after hearing the instructions. During this period, the sEMG signals were collected from 10 muscles of the subject's lower-limbs.

**Figure 2:**
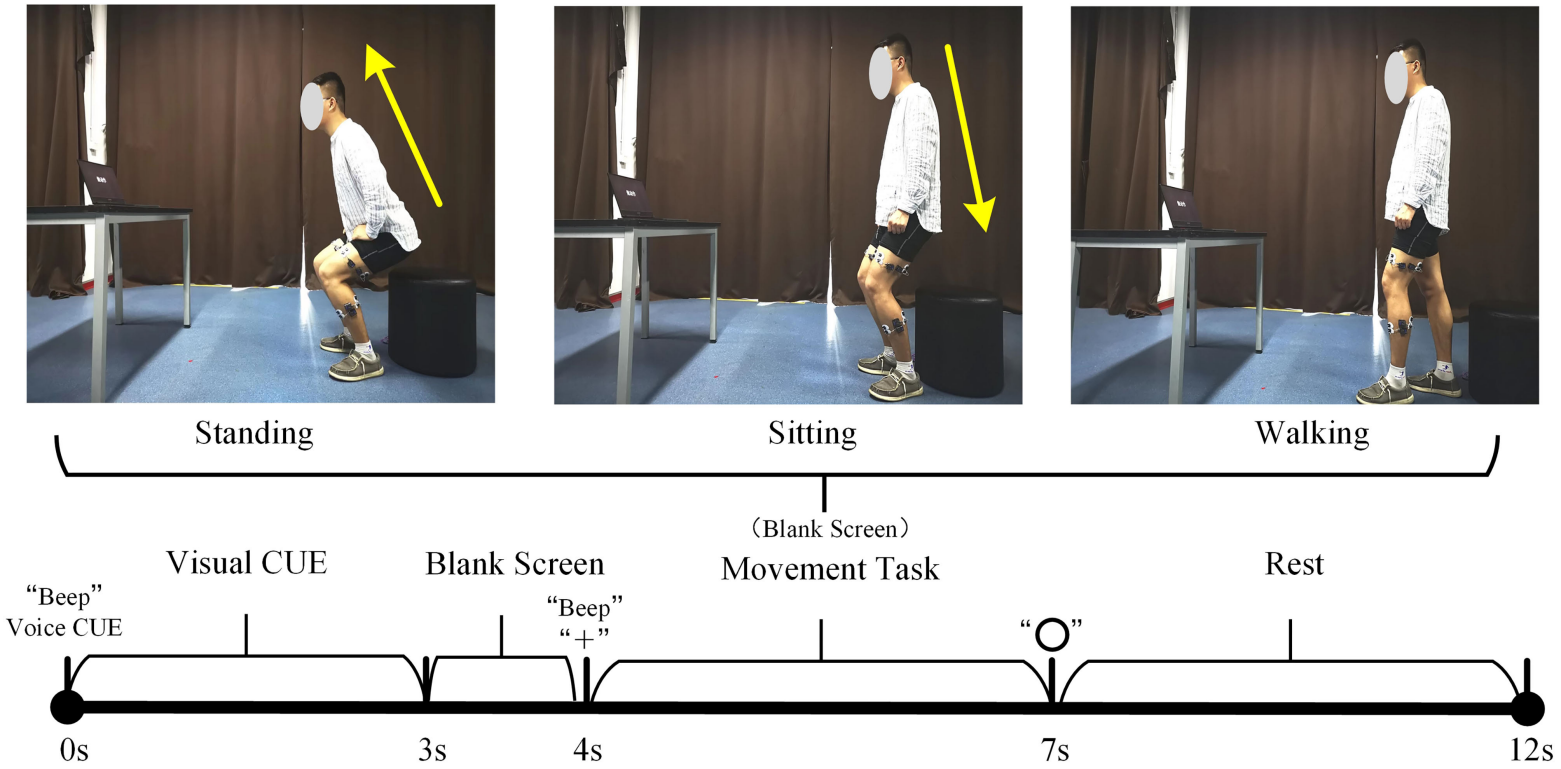
Schematic diagram of sEMG data acquisition experiment. The **upper** part is the preparation posture of the lower limb movements. The **lower** part is the experimental acquisition paradigm.

**Figure 3:**
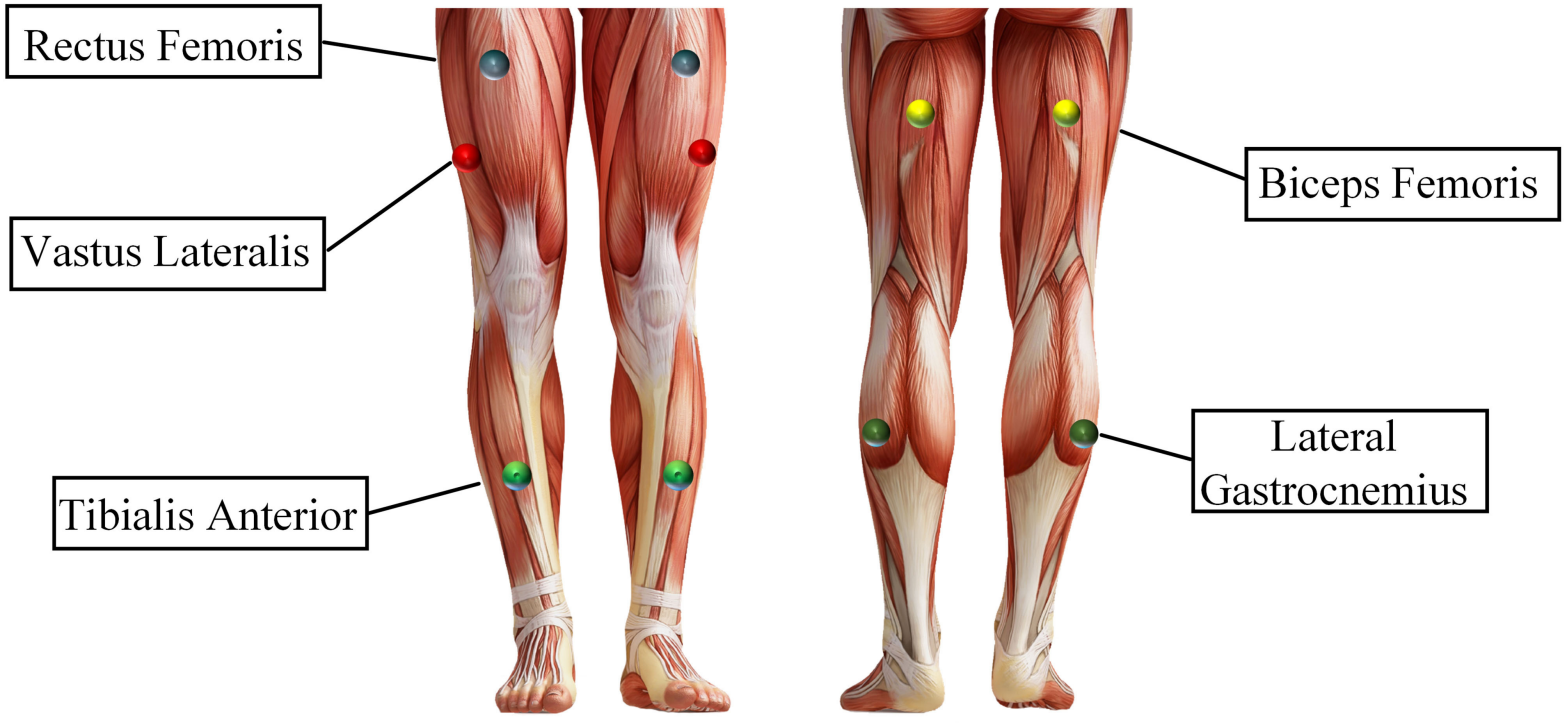
The lower limb muscle used in the sEMG data acquisition experiment.

1. ***Participants***: The 10 subjects (7 men, 3 women) had an average age of 24 years, a height between 162 and 181 *cm*, and a weight between 45 and 80 *kg*. All subjects were in good mental and physical condition, and the lower limbs were not injured before the experiment. Each subject could independently complete the three lower limb movements of standing, sitting, and walking. Before the experiment, each subject was informed of the experimental content and signed an informed consent form. This experiment was approved by the Ethics Committee of Tianjin Medical University.
2. ***Procedures***: Before the experiment, record the relevant physical parameters of the subjects and inform the subjects of the experimental content. Demonstrate the sEMG experimental acquisition paradigm (as shown in Figure 2) to the subjects until the subjects are familiar with the experimental paradigm and perform the corresponding actions within the specified time. Then paste sEMG acquisition electrodes on the 10 muscles of the subject's left and right lower limbs, including the rectus femoris, vastus lateralis, tibialis anterior, biceps femoris, and lateral gastrocnemius of every leg, as shown in Figure 3). During the experiment, the subjects stand in front of the screen and relaxed naturally, and then followed the sEMG acquisition paradigm to complete the corresponding movement, as follows:
  - 0–3 s: A video is played on the computer screen, reminding the subject of the upcoming lower limb movement.
  - 3–4 s: The computer remains blank. At 4 s, the computer will make a “beep”, and a “+” symbol will appear on the screen, reminding the subject to start performing the corresponding lower limb movement.
  - 4–7 s: The subject performs the corresponding lower extremity movements and records the sEMG signals of their lower limbs synchronously.
  - 7–12 s: The “○” pattern appears on the screen, reminding the subject that the single-movement data collection experiment is over. The subjects rest and return to the position and posture at the beginning of this experiment, waiting for the next experiment.

There are three groups in the whole experiment, and each group includes 10 standing, sitting, and walking movements. The standing and walking movements will randomly appear in the first 20 movements, and the last 10 movements are sitting movements (because the subjects are required to remain standing before the standing and sitting movements). During the whole experiment, myoMUSCLE (an sEMG acquisition device, Scottsdale, American) is used to record the subjects' lower-limb sEMG signals.

***Data Processing***:3. myoMUSCLE (1,500 Hz) collects the lower limb sEMG signal data of each lower limb movement of the subject throughout the whole process. After obtaining the sEMG data, a 50 Hz notch filter is used to remove the power frequency interference of the current, and a 10–450 Hz bandpass filter is used to retain the effective information of the sEMG signal. Since the main purpose of this paper is to design an sEMG-based HRI for lower limb motion prediction, we only select the first second sEMG data of the subject's lower limb movement for model training. Considering that the sEMG signal of the affected side of patients with hemiplegia cannot be used, only the sEMG signal of the unaffected lower limb can be collected for movement prediction, so this article only uses the sEMG signal of one side limbs (left side) of all subjects. That is, the number of sEMG channels *C* to be input is 5. In addition, the sliding window size and moving step size of the sEMG signal data will affect the feature extraction and the prediction performance of the model. In order to obtain the optimal sliding window size and moving step size parameters, we designed a parameter comparison experiment. For details, refer to Section 4.3.1.

### 4.2. Implementation Details

After preprocessing the sEMG data, for the traditional hand-crafted feature-based lower limb movement prediction model, use the relevant formula to calculate the features mentioned in Section 3.1.2, and then input the features into the Classification Learner Toolbox to train the prediction model. For the problem of imbalance in the number of samples between movements, we apply a movement class-weight to the loss function. The class-weight we apply is the inverse of the proportion in the training data, with the majority movement class set to 1.

HCSNet and the deep learning-based lower limb movement prediction models are implemented using the PyTorch library (Paszke et al., 2017). Exponential linear units (ELU) (Clevert et al., 2015) are used to introduce the non-linearity of each convolutional layer. To train ours and other deep learning-based models, we use the Adam optimizer to optimize the model's parameters, with the default setting described in Kingma and Ba (2014) to minimize the categorical cross-entropy loss function. We run 1,000 training iterations (epochs) and perform validation stopping, saving the model weights, which produce the lowest validation set loss. All models are trained on NVIDIA RTX2080Ti, with CUDA10.1 and cuDNN V7.6.

### 4.3. Experiments Result

In the experimental results section, we first show the effect of different sliding window parameters on the performance of the lower-limb movement prediction method and determine the sliding window parameters by comparison. Second, we compare HCSNet with other lower-limb movement prediction methods in both within-subject and cross-subject situations.

#### 4.3.1. Sliding Window Parameter Determination

**Figure 4:**
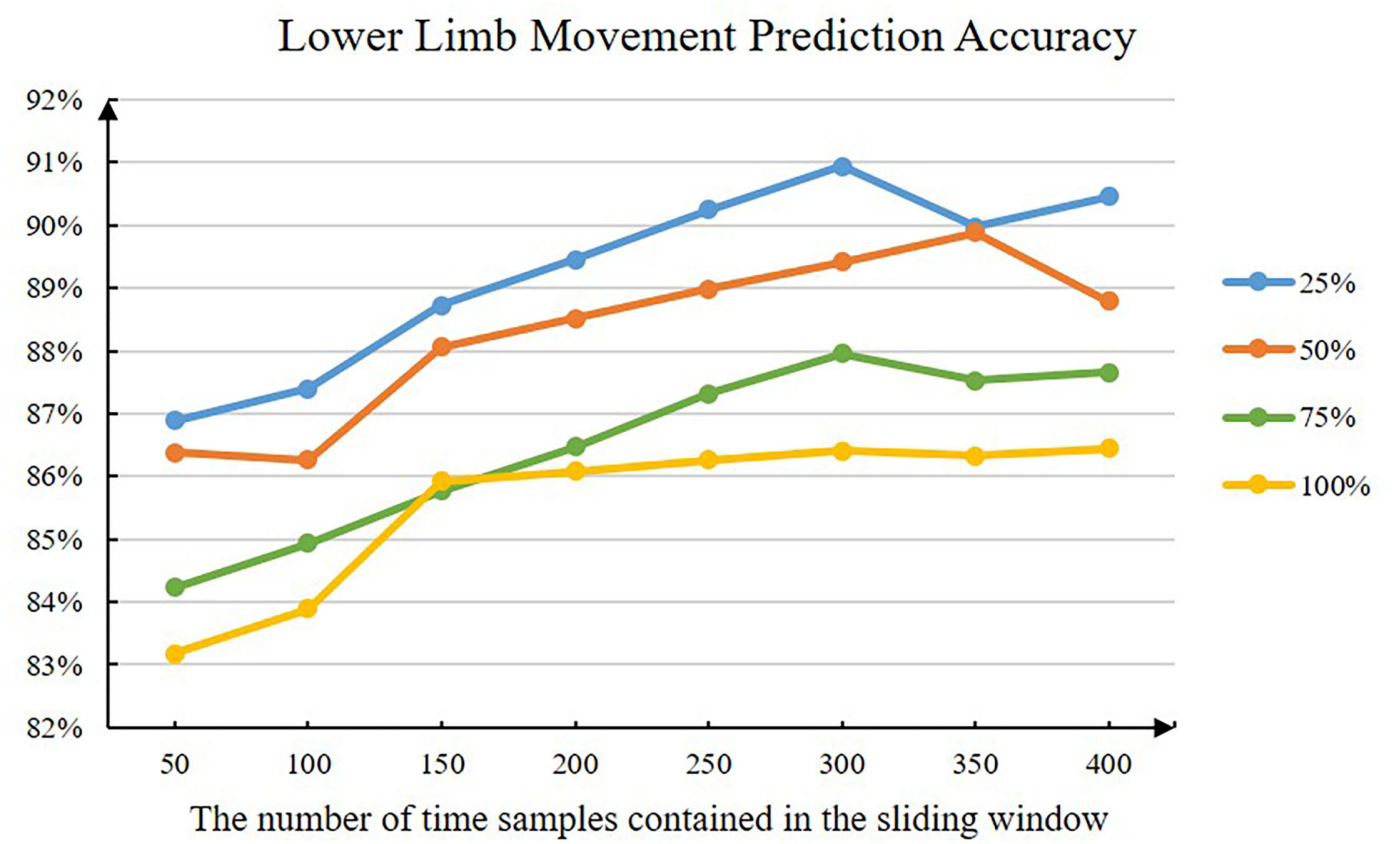
The accuracy of the lower limb movement prediction method when the sliding window parameter has changed. The abscissa represents the number of time samples contained in different sliding windows. The lines of different colors represent different moving step sizes (e.g., 25% means that the moving step size is 25% of the sliding window size).

The size of the sliding window and the moving step length will affect the extraction of sEMG signal features, especially the extraction of hand-crafted features, which in turn affects the performance of the lower-limb movement prediction methods. In this article, a sliding window parameter comparison experiment is designed. By setting different sliding window sizes and moving steps, the changes in the accuracy of the lower limb movement prediction method based on hand-crafted features and machine learning are observed, and the sliding window parameters are determined. The specific results are shown in Figure 4.

Figure 4 shows the average lower limb movement prediction accuracy of all subjects under different sliding window parameters. The model used is a lower limb movement prediction model based on hand-crafted features and RBFSVM. It can be intuitively seen that as the length of the sliding window increases, the prediction accuracy of the model will gradually increase. When the sliding window size is about 300, the prediction accuracy of the model reaches the inflection point. In addition, the smaller the moving step size, the higher the prediction accuracy of the model. Considering the interaction period of the actual HRI, we finally choose the sliding window size as 300 and the moving step as 150 (that is, 50% of the sliding window size).

#### 4.3.2. Within-Subject Classification

For within-subject, we divide the data of the same subject according to a ratio of 7:3 and then use 70% of the data to train the model for that subject. Four-fold cross-validation is used to avoid the phenomenon of model overfitting.

We compare the performance of both hand-crafted features and machine learning-based lower-limb movement prediction models (RBFSVM, LDA, KNN, and RF) and deep learning-based lower-limb movement prediction models (MCSNet, GoogleNet, and ResNet) with HCSNet.

**Figure 5:**
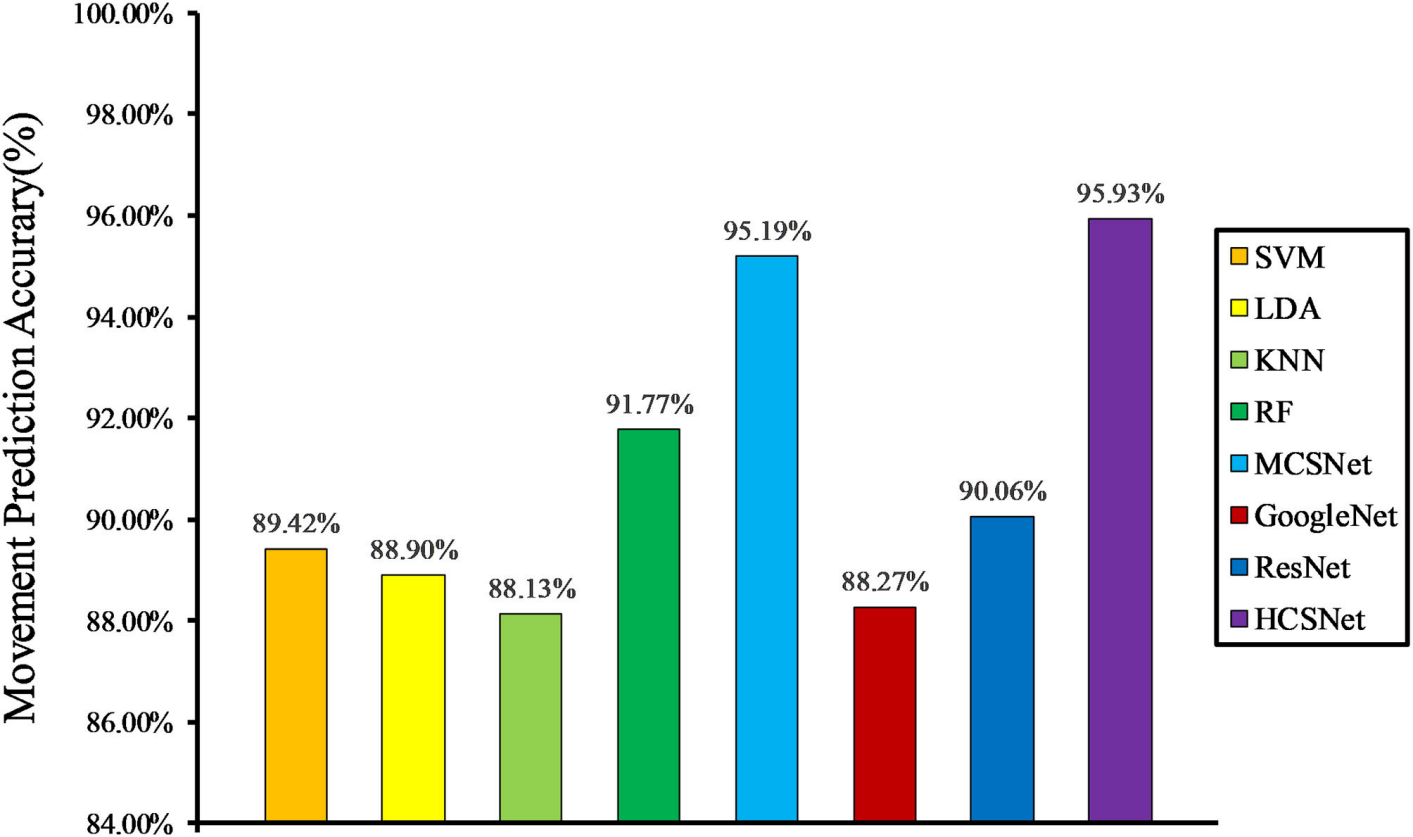
Within-subject movement prediction performance, 4-fold cross-validation is used to avoid the phenomenon of model overfitting averaged over all folds and all subjects.

It can be clearly seen from Figure 5 that after fusing hand-crafted features and learning-based features, the lower-limb movement prediction accuracy of HCSNet is significantly higher than other movement prediction models, especially the models based on handcrafted features. In addition, the lower-limb movement prediction accuracy of GoogleNet and ResNet is close to the lower-limb movement prediction models based on hand-crafted features, which shows that in the case of small samples, the pure deep learning method cannot extract more sEMG information. However, the lower-limb movement prediction accuracy of HCSNet is significantly higher than that of GoogleNet and ResNet, which indicates that the extracted hand-crafted features and the learning-based features have complementary information, and the features learned based on deep learning methods cannot completely replace the hand-crafted features. Table 1 shows the prediction accuracy of each subject under different lower-limb movement prediction models. It can be found that HCSNet can achieve 100% lower-limb movement prediction for most subjects. It means that HCSNet can effectively extract each subject's lower limb movement feature, thereby achieving good movement prediction.

**Table 1:** Within-subject movement prediction performance (Test set ACC).

**Subject ID**	**SVM (%)**	**LDA (%)**	**KNN (%)**	**RF (%)**	**MCSNet (%)**	**GoogleNet (%)**	**ResNet (%)**	**HCSNet (%)**
S1	88.50	87.60	85.80	89.20	100.00	80.99	88.31	100.00
S2	91.70	89.40	89.00	94.20	92.59	90.56	91.23	96.30
S3	96.20	93.90	94.40	96.10	96.30	93.48	94.80	100.00
S4	91.80	92.40	93.20	95.40	100.00	90.87	89.94	100.00
S5	90.10	89.90	90.00	90.80	100.00	86.54	93.38	100.00
S6	81.80	80.10	80.30	85.40	77.78	78.85	79.81	74.07
S7	89.00	89.70	86.50	90.30	100.00	89.29	94.99	100.00
S8	82.10	82.40	82.90	87.90	85.19	84.85	84.30	88.89
S9	91.80	92.90	88.80	94.40	100.00	93.06	94.45	100.00
S10	91.20	90.70	90.40	94.00	100.00	94.22	89.37	100.00
Ave acc	89.42	88.90	88.13	91.77	95.19	88.27	90.06	95.93

#### 4.3.3. Cross-Subject Classification

In the case of cross-subject, we randomly selected the data of seven subjects to train the model and selected the data of three subjects as the validation set. The whole process is repeated five times, producing five different folds.

**Figure 6:**
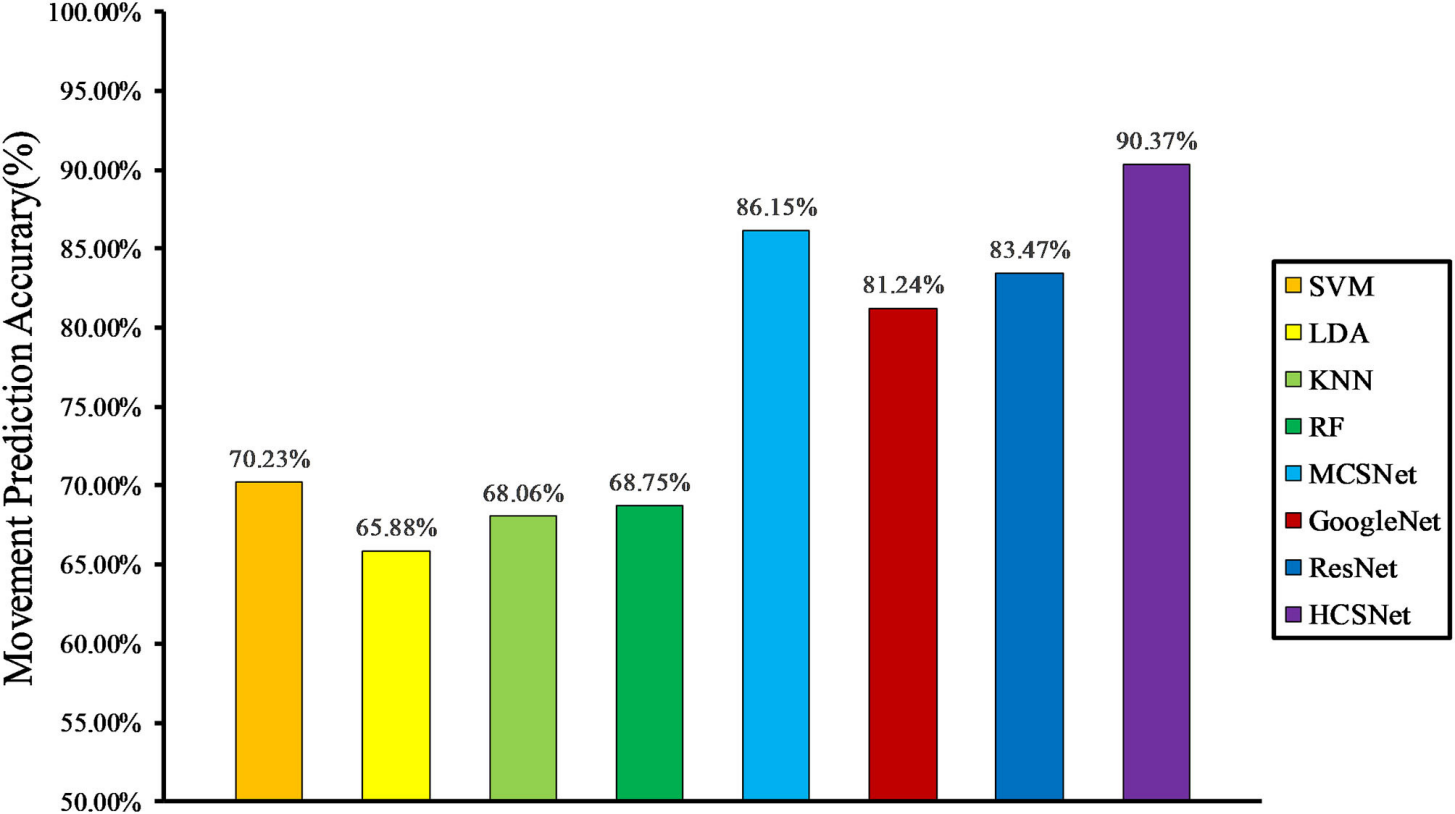
Cross-subject movement prediction performance averaged over all folds.

Cross-subject prediction results across all models are shown in Figure 6. It can be seen that the hand-crafted features-based lower-limb movement prediction models have poor performance in the cross-subject situation, with an average accuracy rate of about 70%. In addition, the lower-limb movement prediction model based on learning-based features outperforms the models based on hand-crafted features. This shows that in the case of big data, hand-crafted features contain less effective information than learning-based features. In this case, the HCSNet model proposed in this paper can still achieve an accuracy of 90.37% in lower limb movement prediction, which is far from other models. It proves the effectiveness of HCSNet.

## 5. Conclusion

In this article, a human-exoskeleton interface for lower limb movement prediction in patients with hemiplegia is proposed. It uses the sEMG signals of the unaffected lower limbs of patients with hemiplegia to predict lower-limb movements and constructs a lower limb movement prediction framework (HCSNet) that fuses hand-crafted features and deep neural networks. The synergy features of different sEMG channels are extracted using MCSNet, which are subsequently combined with hand-crafted features such as time and frequency domains. An sEMG data acquisition experiment is designed to verify the proposed HCSNet. In the two cases of within-subject and cross-subject, the lower limb movement prediction performance of HCSNet is compared with four traditional machine learning-based movement prediction models and three state-of-the-art deep learning-based movement prediction models. The results show that HCSNet has a good movement prediction performance in both within-subject and cross-subject situations. In the future, we consider applying the proposed human-exoskeleton interface to an actual exoskeleton platform. In addition, we will focus on multi-modal movement prediction based on sEMG and EEG.

## Data Availability Statement

The raw data supporting the conclusions of this article will be made available by the authors, without undue reservation.

## Ethics Statement

The studies involving human participants were reviewed and approved by the Ethics Committee of Tianjin Medical University. The patients/participants provided their written informed consent to participate in this study.

## Author Contributions

XY designed the movement prediction model, performed the experiments, and drafted the manuscript. BL and JL participated in the design of the movement prediction model and assisted in the manuscript writing. ZF guided writing manuscripts and doing experiments. All authors contributed to the article and approved the submitted version.

## Conflict of Interest

The authors declare that the research was conducted in the absence of any commercial or financial relationships that could be construed as a potential conflict of interest.

## Publisher's Note

All claims expressed in this article are solely those of the authors and do not necessarily represent those of their affiliated organizations, or those of the publisher, the editors and the reviewers. Any product that may be evaluated in this article, or claim that may be made by its manufacturer, is not guaranteed or endorsed by the publisher.
